# First identification of *Rickettsia helvetica* in questing ticks from a French Northern Brittany Forest

**DOI:** 10.1371/journal.pntd.0005416

**Published:** 2017-03-01

**Authors:** Sarah I. Bonnet, Richard E. L. Paul, Emmanuel Bischoff, Martine Cote, Evelyne Le Naour

**Affiliations:** 1 UMR BIPAR INRA-ANSES-ENVA, Maisons-Alfort cedex, France; 2 Institut Pasteur, Unité de Génétique Fonctionnelle des Maladies Infectieuses, Paris, France; 3 CNRS URA3012, Paris, France; 4 Institut Pasteur, Unité de Génétique et génomique des insectes vecteurs, Paris, France; Mahidol University, THAILAND

## Abstract

Tick-borne rickettsiae are considered to be emerging, but data about their presence in western Europe are scarce. *Ixodes ricinus* ticks, the most abundant and widespread tick species in western Europe, were collected and tested for the presence of several tick-borne pathogens in western France, a region never previously explored in this context. There was a high tick abundance with a mean of 4 females, 4.5 males, and 23.3 nymphs collected per hour per collector. Out of 622 tested ticks, specific PCR amplification showed the presence of tick symbionts as well as low prevalence of *Borrelia burgdorferi* (0.8%), *Bartonella* spp. (0.17%), and *Anaplasma phagocytophilum* (0.09%). The most prevalent pathogen was *Rickettsia helvetica* (4.17%). This is the first time that this bacteria has been detected in ticks in this region, and this result raises the possibility that bacteria other than those classically implicated may be involved in rickettsial diseases in western France.

## Author summary

Due to socio-economical and meteorological changes, the geographical distribution of several tick species is changing. As ticks are the most important vectors of pathogens in northern latitudes, performing epidemiological studies is essential to assess the extent of tick activity as well as the risk of pathogens transmission. The detection of the bacteria *Rickettsia helvetica* in questing ticks in western France, for the first time, raises the possibility that bacteria other than those classically implicated may be involved in rickettsial diseases in this region.

## Introduction

Ticks are one of the most important infectious disease vectors worldwide, and are second only to mosquitoes in the frequency of human pathogen transmission [1]. They are obligate blood feeding arthropods and transmit the largest variety of pathogens. Currently, the emergence of tick-borne diseases (TBD) is a growing concern, and their incidence is on the rise in many European countries favored by both socio-economic and environmental changes, and highlighting the need to increase surveillance of tick populations and associated pathogens [2–5]. Lyme disease caused by *Borrelia* species is unquestionably the predominant concern for the northern latitude [1]. However, ticks, and in particular *Ixodes ricinus* in Europe, which frequently bites humans, can transmit a large variety of other potentially dangerous human pathogens [3] including rickettsiae [6]. Rickettsiae are obligate intracellular alpha-proteobacteria distributed worldwide, and transmitted to humans and animals via arthropod vectors including insects, as well as ticks and mites [7]. Ticks are known vectors of rickettsiae responsible for spotted fever syndrome in humans, which is caused by at least 15 different Rickettsia species. The most life-threatening species are *R*. *rickettsia*, the agent of Rocky Mountain spotted fever and *R*. *conorii*, the causative agent of Mediterranean spotted fever, but several species of tick-borne rickettsiae that were considered non-pathogenic for decades are now associated with human infections [7].

Among potential emerging rickettsia species, *Rickettsia helvetica* is considered as an emerging tick-borne pathogen (TBP), and has been first recognized in 1979 in *I*. *ricinus* as a new member of the spotted fever group of *Rickettsia* [8]. Later, in eastern France, following a human febrile infectious syndrome with specific seroconversion against *R*. *helvetica*, a 9.2% seroprevalence rate was reported in humans exposed to tick bites [9]. The bacteria has also been isolated from *I*. *ricinus* in central France, confirming its presence in this region, as suspected from a previous seroprevalence survey [10]. Since, *R*. *helvetica* has been associated with two cases of fatal perimyocarditis in Sweden, as the bacteria was detected by both Polymerase Chain Reaction (PCR) and immunohistochemistry in the pericardium, the pulmonary hilum, coronary artery and the heart muscle [11]. The bacteria has also been detected by PCR in samples obtained from two dead patients with sarcoidosis, and immunohistochemical examination showed presence of rickettsia-like organisms, suggesting that it may contribute to the granulomatous process, as is seen in sarcoidosis [12]. Antibodies against *R*. *helvetica* have been also associated with febrile illness after tick bite in several European and South-East Asian countries [13], where immunohistochemical examination has confirmed the presence of the bacteria [14]. Lastly, in addition to positive serology, *R*. *helvetica* has been detected by PCR in two patients with acute febrile illness, rash and long-lasting myasthenia [15], and subacute meningitis [16].

In Europe, *R*. *helvetica* is strongly suspected to be transmitted by *I*. *ricinus*, being detected in ticks in several European countries [14,17–28]. To confirm vector transmission, competence studies under controlled conditions are required. However, indirect proof of tick vector competency has been reported through detection of anti-*R*. *helvetica* antibodies in people exposed to tick bites [29]. In addition, the detection of *R*. *helvetica* in engorged *I*. *ricinus* found on unifected hosts as well as vertical transmission in ticks, strongly suggest this tick species is a reservoir of this pathogen [30,31]. Due to the broad host range of *I*. *ricinus*, many vertebrate species may also serve as potential reservoirs for the bacteria. *R*. *helvetica* has been found in blood from mice, wild rodents, roe deer, and wild boar, all without clinical signs of infection [23,30], suggesting a zoonotic cycle, in which humans represent recent and accidental hosts. In the Netherlands, 24.7% of *I*. *ricinus* ticks collected from domestic animals were found to be infected with *R*. *helvetica* [19] when in Switzerland, 50% and 28% of ticks collected from cats and dogs respectively, were positive [32]. Interestingly, these infection rates were higher in ticks collected from these animals than in ticks collected from vegetation in the same region. A similar result was observed for ticks collected from roe deer, dogs and birds elsewhere in Europe [33–36]. Altogether, these results suggest that both domestic and wild animals may act as reservoirs for *R*. *helvetica* transmitted by *I*. *ricinus*.

Fastidious epidemiological studies are still required in order to have a better understanding of the geographical distribution of TBP, and to increase public awareness of the potential danger represented by ticks. The aim of the present study was to obtain an overall picture of potentially high-risk TBP circulating in northern Brittany in western France, a region never previously explored in this context.

## Materials and methods

### Study area, spatial data and maps

**Fig 1:**
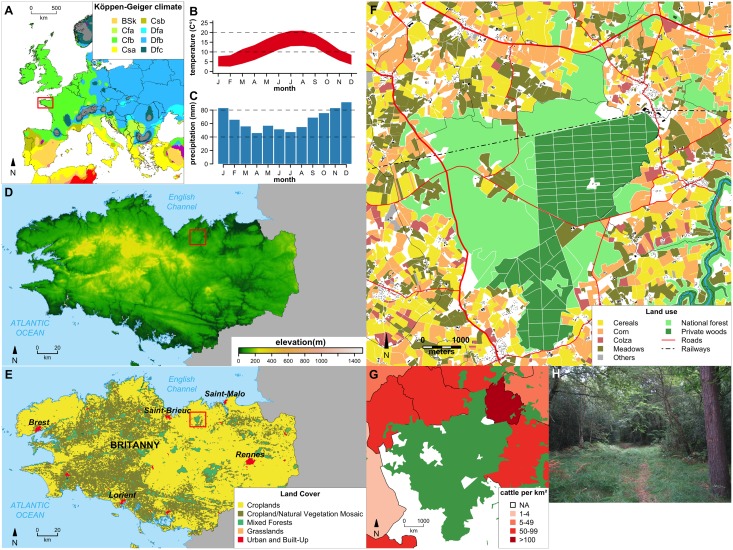
Detailed description of the sampling site. (A) European map of the of Köppen-Geiger climate classification (from Peel et al, 2007). Location of Brittany is indicated by a red square. (B) Average monthly minimum and maximum temperatures for the La Hunaudaye Forest (from Hijmans, 2005). (C) Average monthly precipitations for the La Hunaudaye Forest (from Hijmans, 2005). (D) Brittany elevation map (from Jarvis et al. 2008). Location of La Hunaudaye Forest is indicated by a red square. (E) Brittany land cover (from Broxton et al. 2014). Location of La Hunaudaye Forest is indicated by a red square. (F) Detailed map of La Hunaudaye Forest and its vicinity with crop types. (G) Cattle per km. (H) Picture showing the ecological facies of the sampling site.

Ticks were collected in May 2014 in the “forêt de la Hunaudaye” (10.40km^2^, 48.482945 N; 2.365779 W), Côtes d’Armor, Brittany, western France, located 15 km from the sea (Fig 1A and 1D). Given that tick abundance is influenced by climate, vegetation, elevation, and host presence and densities, a complete description of these factors in the studied area was made to enable comparison with current and future studies (Fig 1). All maps were created using the WGS84 coordinate reference system; shapefiles were converted if needed. All spatial and geographical data were processed with R [37]. The climate map, created using data of the Köppen-Geiger climate classification [38], showed a temperate climate with warm summer (Fig 1B) without dry season (Fig 1C). Average monthly minimum and maximum temperatures and precipitation were downloaded from the WorldClim website (version 1.4; http://www.worldclim.org) with a resolution of 30 arc-seconds [39]. SRTM 90m Digital Elevation Data were downloaded from the CIAT-CSI SRTM website [40] and elevation ranged from 71–112 meters across the forest (Fig 1D). Land cover data were taken from Broxton et al. [41]. Wood areas, rivers, roads, railway and buildings spatial data were downloaded from OpenStreetMap website (http://www.openstreetmap.org). Crop types per plot were taken from the "Registre Parcellaire Graphique Bretagne 2013 Contours des îlots culturaux et leur groupe de culture majoritaire", downloaded from GéoBretagne website (*geobretagne*.*fr/geonetwork/srv/fre/pdf?id=18162*). The study site is mainly covered by croplands, natural vegetation and mixed forests (Fig 1E), populated by beech (*Fagus sylvatica)* and oak (*Quercus sp*.*)* with conifers and holly (*Ilex aquifolium)*. The forest massif is comprised of both state forest and private plots representing an overall surface of 25.98 km^2^ (Fig 1F). Fauna populating the forest include deer (*Cervus elaphus)*, roe deer (*Capreolus capreolus)*, wild boar (*Sus scrofa)*, diverse rodents, and birds. This forest is situated in a rural area and is surrounded by cattle farms and cultivated land (Fig 1F, 1G and 1H). Number of cattle per district was extracted from 2010 agricultural census data (Agreste database, data.gouv.fr) and were normalized by district surface. Thanks to numerous paths enabling recreational activities, the forest is highly frequented by walkers with also a lot of hunting activity, including hunting with hounds.

### Tick collection

Questing ticks (nymphs and adults) were collected using the flagging method, whereby 1 m^2^ cotton cloths are dragged over the vegetation, from 16:30 to 19:30 on the 24^th^ of May 2014 by four collectors and from 15:30 to 18:00 on the 25^th^ by three collectors. The weather varied between overcast and sunny, ground vegetation remained wet, and the temperature remained 17–18°C. Tick activity was estimated as number of ticks per collector per hour, as previously calculated [17]. All specimens, returned alive to the laboratory, were then identified to the species level using taxonomic keys, categorized by sex and life stage, and frozen at -20°C prior to DNA extraction.

### DNA extraction

Ticks were crushed, individually for adults and in pools of five for nymphs, by shaking with a bead beater (mixer mill MM301, Qiagen, Hilden, Germany) as previously described [42]. DNA was extracted using the Nucleospin Tissue kit according to the manufacturer’s instructions (Macherey-Nagel, Duren, Germany). Adults and nymph pools were eluted in a final volume of 50 μL. DNA extracts were then stored at -20°C until use. DNA extraction efficiency was confirmed in all samples with polymerase chain reaction (PCR) amplification of the 16S rRNA mitochondrial gene using tick-specific primers TQ16S+1F (5′-CTGCTCAATGATTTTTTAAATTGCTGTGG-3′) and TQ16S-2R (5′-ACGCTGTTATCCCTAGAG-3′), as described [43].

### Polymerase chain reaction amplification

Specific PCRs were used to detect the presence of *B*. *burgdorferi* s.l., *Anaplasma* spp./*Candidatus Midichloria mitochondrii/Wolbachia* spp., SFG *Rickettsia* spp., *Babesia/Theileria* spp., *F*. *tularensis* and *Bartonella* spp. DNA in tick extracts as previously described [42]. All PCR reactions were performed in a MyCycler thermocycler (Bio-Rad, Strasbourg, France). Each reaction was carried out in a 25 μL volume containing 0.5 μmol/μL of each primer, 2.5 mmol/L of each dNTP, 2.5 μL of 10X PCR Buffer, 1U of Taq DNA polymerase (Takara Biomedical Group, Shiga, Japan), and 5 μL of each DNA extract. Negative (sterile water) and positive DNA controls were included in each run as previously described [42].

### Sequencing and sequence analysis

Qiagen (Hilden, Germany) performed sequencing on all positive samples, either directly on the PCR product or following extraction from agarose gel and purification using the NucleoSpin Extract II kit (Macherey-Nagel, Duren, Germany). Sequences obtained were compared with known sequences listed in the GenBank nucleotide sequence databases via the National Center for Biotechnology Information Blast search option (www.ncbi.nlm.nih.gov/BLAST), and sequence data were deposited in GenBank.

### Statistical analysis

Prevalence rates and exact binomial 95% confidence intervals were independently calculated for each microorganism in male and female adult ticks using Ecological Methodology software [44]. Prevalence rates were compared between males and females with the Fisher Exact test, using Genstat version 15 (VSN International Ltd., Hemel Hempstead, UK). For the pooled nymph samples, we employed the exact method of Hauck, assuming perfect sensitivity and specificity of our pathogen detection methods [45]. Hauck noted a one-to-one relationship between individual level prevalence, π, and the prevalence of positive pools, *P*. A point estimate for the prevalence rate can thus be obtained from the pool positive rate by π = 1-(1-*P*)^1/*k*^ where k is the number of nymphs per pool. Exact 95% confidence intervals were then obtained by assuming a binomial distribution for the number of positive pools [46]. Nymph and adult female and/or male samples were then compared and considered to be significantly different if there was no overlap in 95% confidence intervals. In addition, the estimated nymph prevalence rates were used to estimate the number of individual nymphs infected. Prevalence rates of nymphs and adult ticks were then compared with the Fisher Exact test.

### Accession numbers

The obtained sequences were submitted to Genbank with the following accession numbers: *A*. *phagocytophilum*: KU559922; *R*. *helvetica*: KU559920; and *C*. *Midichloria mitochondrii*: KU559921.

## Results

### Tick collection and efficiency of DNA extraction

A total of 622 ticks were collected from the vegetation, of which all were identified as *I*. *ricinus*. The collection comprised 78 females, 89 males, and 455 nymphs, which corresponded to 4 females, 4.5 males, and 23.3 nymphs collected per hour per collector. DNA was extracted from 258 samples: 91 pools with 5 nymphs each, and 167 single adults. The *I*. *ricinus* 16S rRNA gene was amplified in 231/258 samples (90%), which were then included in the study. No amplification products were obtained for 27 samples, corresponding to 18 females, 4 males, and 5 pools of nymphs, reflecting a probable failure of the DNA extraction, and were thus excluded from the analysis.

### Detection of microorganisms carried by ticks

PCR detection results are presented in Table 1. Sequencing of *Anaplasma* spp. positive samples revealed only one pool of nymphs positive for *Anaplasma phagocytophilum*, whereas the remaining positive samples indicated the presence of the tick symbiont, *C*. *Midichloria mitochondrii*. The estimated point prevalence in nymphs of *C*. *Midichloria mitochondrii* and *A*. *phagocytophilum* was 13.4% and 0.2% respectively, with an overall prevalence in all ticks of 11.3% and 0.09% respectively. *C*. *Midichloria mitochondrii* prevalence rates were significantly higher in adult females (16.7%) than males (4.7%) (P = 0.021), whereas the estimated point prevalence in nymphs was not different to rates observed in adult females and males (overlapping 95% confidence intervals, Fisher Exact p-value = 0.52 nymphs in comparison to adult females, and P = 0.068 with adult males).

**Table 1:** Prevalence and 95% binomial exact confidence intervals of *Ixodes ricinus* ticks harboring selected tick-borne pathogen DNA. π is a point estimate for the prevalence rates in nymph pools (see Methods). Overall prevalence is the mean of adult female, male, and estimated nymph prevalence rates.

*Ixodes ricinus* samples	Number of infected ticks (prevalence (%)) (binomial 95% confidence intervals)							
	number of analyzed samples	*C*.*M*. *mitochondrii*	*A*. *phagocytophilum*	*Rickettsia* spp.	*Borrelia burgdorferi* s.l.	*Bartonella* spp.	*Babesia-Theileria* spp.	*Francisella tularensis*
**Females**	60	**10 (16.7)**	0	0	0	0	0	0
		(8.3–28.5)	(0–6.0)	(0–6.0)	(0–6.0)	(0–6.0)	(0–6.0)	(0–6.0)
**Males**	85	**4 (4.7)**	0	**9 (10.6)**	**2 (2.4)**	0	0	0
		(1.3–11.5)	(0–4.2)	(4.9–18.9)	(0.3–8.1)	(0–4.2)	(0–4.2)	(0–4.2)
**Nymphs (pools)**	86	**44 (51.2)** (40.1–62.1)	**1(1.2)** (0–6.3)	**8 (9.3)** (4.1–17.5)	0 (0–4.2)	**2 (2.3)** (0.3–8.2)	0 (0–4.2)	0 (0–4.2)
**Nymphs Estimated prevalence, π**		**13,4** (9.8–17.6)	**0,2** (0–1.3)	**1.9** (0.8–3.8)	0 (0–0.9)	**0.5** (0.1–1.7)	0 (0–0.9)	0 (0–0.9)
**Overall prevalence (%)**		**11.3**	**0.09**	**4.17**	**0.80**	**0.17**	0	0

C.M. mitochondrii: Candidatus Midichloria mitochondrii; A. phagocytophilum: Anaplasma phagocytophilum.

In contrast, the percentage of *Rickettsia* spp. positive samples was significantly higher in adult males (10.5%) than either females (0%) (P = 0.007) or nymphs (1.9%, non-overlapping 95% confidence intervals, Fisher’s Exact p-value = 0.032). Sequencing analysis demonstrated that all amplified sequences corresponded to *R*. *helvetica* with 100% identity with sequences present in databanks. *B*. *burgdorferi* and *Bartonella* spp. infection prevalence rates were very low, with no differences between adult males, females, and nymphs. Unfortunately, we were unable to sequence the corresponding amplicons for these two genera. No positive samples of *Babesia*, *Theileria*, or *Francisella* spp. were identified.

## Discussion

Relatively few epidemiological surveys have explored simultaneously the presence of multiple emerging human tick-borne pathogens considered to be important in France, as well as in Europe generally. To determine the presence of such pathogens in a french western region never previously investigated, 622 *I*. *ricinus* ticks were collected and screened for DNA of pathogens in a typical recreational Brittany forest. Tick abundance was higher than those previously obtained from the Sénart forest near Paris, France [17]. Thus this high tick abundance justifies increased surveillance for those TBP that could be transmitted to humans.

Firstly, we detected the presence of C. *Midichloria mitochondrii*, an intra-mitochondrial symbiont bacterium detected in several tick genera including *Ixodes* spp. [47]. This bacterium may have a possible helper role in tick molting processes [48], and despite believed to be harmless to mammals, it was recently suggested that it can be pathogenic for some vertebrate hosts [49], and may have possible roles in the transmission of other tick-borne pathogens [47]. As for the known TBP, *A*. *phagocytophilum* was detected in only one nymph pool, leading to an overall prevalence of 0.09%, reflecting reported rates in France [17,50–53]. *B*. *burgdorferi* s.l. had an overall low prevalence (0.8%), similar to some rates previously reported in France, which can varied from 0 to 29% [17,51,53,54]. The absence of *Babesia* sp. in the study area was surprising considering the proximity of numerous bovine herds, which could act as *Babesia divergens* reservoirs (Fig 1G) [55], and the presence of roe deer (promoted by arable farmed areas) in the forest, believed to be *Babesia venatorum* parasite reservoirs [56].

Regarding the increasing numbers of reports on the pathogenicity of *R*. *helvetica* in humans, the most significant result of the present study was the relatively high *R*. *helvetica* prevalence rate of 4.17% in questing *I*. *ricinus*. This is higher than the rate previously observed in 2006 in ticks from another area in western France 150 km from the current area (1.4%) [53], but is similar to rates reported near Paris (France) in 2008 [17]. In an extensive study evaluating the occurrence of *Rickettsia* spp. in the Netherlands from 2000 to 2008, Sprong et al. reported prevalence rates from 6% to 66% in ticks depending on location, emphasizing the heterogeneous but increasing and persistent presence of this bacterium in Europe [23]. Indeed, the reported occurrence of this bacterium in ticks has varied from 3–14% in other European countries [18,57]. The recent reports presenting evidence of *R*. *helvetica* bacteraemia in birds, including migratory species, as well as *R*. *helvetica* presence in bird ticks, highlight the danger represented by avian populations for both enzootic maintenance and potentially vast distribution zones of the bacteria and infected ixodid ticks throughout Europe [33–35].

It was surprising that *R*. *helvetica* was not detected in female ticks in our study, when in the Netherlands, Sprong et al found no differences between tick life-stages [23], and when usually infection prevalence in questing adults ticks exceeded infection rates in questing nymphs [58]. This discrepancy may suggest lowered transtadial transmission efficiency between nymphs and females and/or influence of tick microbiomes that may differ between tick life stages, and requires further investigation. Given that vertical bacterial transmission has been demonstrated in ticks under laboratory conditions, we should perhaps reconsider whether *R*. *helvetica* is predominantly a tick symbiont rather than a pathogen; this again highlights—for rickettsiae in particular and tick-borne microorganisms in general—the fine line between pathogenic and symbiont status [31].

Our findings contribute further knowledge to the geographic distribution of the studied pathogens, and to the significant risks of infection in people exposed to *I*. *ricinus* ticks, including *R*. *helvetica*, considered as an emerging TBP able to infect humans. Our results confirm *R*. *helvetica*’s reported wide distribution in Europe, emphasize that *R*. *helvetica* infection must be considered when diagnosing patients bitten by ticks in western France, where, although Lyme disease is now a recognized public health issue, it is not the case for the other TBD, such as the rickettsioses. Further studies are now required to improve pathogen characterization, to clarify *R*. *helvetica*’s pathogenicity in humans, and to evaluate the role of ticks as reservoirs and in the spread of infection.
